# Enhancing the Expression of the *OsF3H* Gene in *Oryza sativa* Leads to the Regulation of Multiple Biosynthetic Pathways and Transcriptomic Changes That Influence Insect Resistance

**DOI:** 10.3390/ijms232315308

**Published:** 2022-12-04

**Authors:** Rahmatullah Jan, Sajjad Asaf, Lubna, Saleem Asif, Eun-Gyeong Kim, Yoon-Hee Jang, Nari Kim, Ahmed Al-Harrasi, Gang-Seob Lee, Kyung-Min Kim

**Affiliations:** 1 Department of Applied Biosciences, Graduate School, Kyungpook National University, Daegu 41566, Republic of Korea; 2 Coastal Agriculture Research Institute, Kyungpook National University, Daegu 41566, Republic of Korea; 3 Natural and Medical Science Research Center, University of Nizwa, Nizwa 611, Oman; 4 Department of Botany, Garden Campus, Abdul Wali Khan University, Mardan 23200, Pakistan; 5 Department of Agricultural Biotechnology, National Institute of Agricultural Sciences, Rural Development Administration, Jeonju 54874, Republic of Korea

**Keywords:** *Oryza sativa*, pathogen, defense system, salicylic acid, jasmonic acid, transcriptomics

## Abstract

The white-backed planthopper (WBPH) is a major pest of rice crops and causes severe loss of yield. We previously developed the WBPH-resistant rice cultivar “*OxF3H*” by overexpressing the *OsF3H* gene. Although there was a higher accumulation of the flavonoids kaempferol (Kr) and quercetin (Qu) as well as salicylic acid (SA) in *OxF3H* transgenic (*OsF3H* or Trans) plants compared to the wild type (WT), it is still unclear how Os*F3H* overexpression affects these WBPH resistant-related changes in gene expression in *OxF3H* plants. In this study, we analyze RNA-seq data from *OxF3H* and WT at several points (0 h, 3 h, 12 h, and 24 h) after WBPH infection to explain how overall changes in gene expression happen in these two cultivars. RT-qPCR further validated a number of the genes. Results revealed that the highest number of DEGs (4735) between the two genotypes was detected after 24 h of infection. Interestingly, it was found that several of the DEGs between the WT and *OsF3H* under control conditions were also differentially expressed in *OsF3H* in response to WBPH infestation. These results indicate that significant differences in gene expression between the “*OxF3H*” and “WT” exist as the infection time increases. Many of these DEGs were related to oxidoreductase activity, response to stress, salicylic acid biosynthesis, metabolic process, defense response to pathogen, cellular response to toxic substance, and regulation of hormone levels. Moreover, genes involved in salicylic acid (SA) and ethylene (Et) biosynthesis were upregulated in *OxF3H* plants, while jasmonic acid (JA), brassinosteroid (Br), and abscisic acid (ABA) signaling pathways were found downregulated in *OxF3H* plants during WBPH infestation. Interestingly, many DEGs related to pathogenesis, such as *OsPR1*, *OsPR1b*, *OsNPR1*, *OsNPR3,* and *OsNPR5,* were found to be significantly upregulated in *OxF3H* plants. Additionally, genes related to the MAPKs pathway and about 30 *WRKY* genes involved in different pathways were upregulated in *OxF3H* plants after WBPH infestation. This suggests that overexpression of the *OxF3H* gene leads to multiple transcriptomic changes and impacts plant hormones and pathogenic-related and secondary-metabolites-related genes, enhancing the plant’s resistance to WBPH infestation.

## 1. Introduction

Rice provides a major portion of food to the world population. Due to biotic stress, a huge amount of rice yield is lost annually, of which 25% is attributed to insect pests [1]. In Asian countries, the white-backed planthopper (WBPH), *Sogatella furcifera* is one of the most economically important oligophagous plant feeders among insect pests [2]. WBPH is a long-distance migratory pest and damages the rice plant by direct feeding it or by acting as a vector and transmitting disease-causing plant viruses [3]. WBPH is a sympatric species occurring in almost all rice varieties and colonizes the crop during the vegetative stage [4]. Due to its phloem-feeding nature, the WBPH sucks nutrients from the stem, resulting in slow growth, leaf yellowing, and severe damage at the seedling stage, causing hopper burn and leading to plant death [5].

In recent decades, pesticides have been used as an effective tool to control the WBPH. Subsequently, the inappropriate use and misuse of pesticides led to the major crises of pesticide resistance, as WBPH has an outstanding capability to improve resistance to a variety of insecticides [6,7]. In 1980s, studies were started to identify and characterize the rice cultivars resistant to WBPH [8]. So far, about 19 major genes and 75 QTLs that confer resistance to WBPH have been identified [9]. In 2014, Ramesh et al. reported 12 major genes resistant to WBPH; none were cloned and further characterized [10]. Our previous study evaluated that overexpression of the *F3H* gene showed resistance to WBPH [11]. *F3H* is one of the key genes of the flavonoid biosynthesis pathway and can significantly regulate the accumulation of flavonoids. Our previous study focused on enhancing the biosynthesis of flavonoids, such as kaempferol and quercetin, through overexpression of the *F3H* gene in response to WBPH stress. 

It has been shown by studies that flavonoid biosynthesis is induced by external constraints, such as pathogens, wounds, UV radiation, phytohormones, and temperature [12]. An increasing body of signal indicates that flavonoid accumulation is strongly associated with phytohormone signals in plant growth. Flavonoids such as kaempferol, quercetin, and apigenin increase localized auxin as they inhibit auxin transporter in the plants [13]. It is reported that ethylene has a repressed function similar to auxin and modulates flavonoids in response to gravity [14]. Additionally, ABA signaling induces the biosynthesis of flavonoids through *HY5* transcription factor, which further mediates flavonol synthase expression [15]. A recent study determined that flavonoids may regulate the ABA-signaling network by detoxifying reactive oxygen species and enhancing ABA biosynthesis [16]. *TT8* is a transcriptional factor involved in regulating the flavonoid pathway and induces jasmonic acid and brassinosteroid biosynthesis [17]. These inferences indicate a close connection between flavonoid and hormone crosstalk. Hence, these limited data provide little information that *F3H* overexpression could transcriptionally regulate hormonal and other defense pathways against WBPH stress. However, RNA-seq analysis can provide a fascinating, unprecedented opportunity for novel gene discovery and other defense pathways against WBPH stress.

In this study, we performed RNA-seq analysis using Illumina NovaSeq 6000 (Illumina, San Diego, CA, USA) to conduct an in-depth sequencing of *OxF3H* (*F3H*) gene overexpressor line of nagdong cultivar (published in [11]) and nagdong wild-type plants after WBPH infestation at different time points (0 h, 3 h, 12 h, and 24 h). This study aims to elucidate transcriptomic responses to the overexpression of the *F3H* gene (*OxF3H*) and predict the overall performance of *OxF3H* transgenic rice plants. The analysis provides a comprehensive overview of the differentially expressed genes (DEGs) associated with WBPH resistance, phytohormones, metabolic pathways, and pathogenesis-related expression in *OxF3H* plants. We also verified these results through quantitative real-time PCR (qRT-PCR).

## 2. Results and Discussion

**Figure 1:**
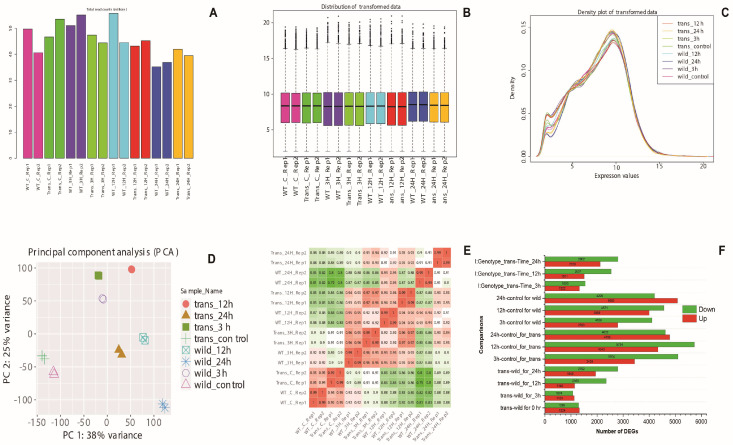
(**A**) Total read count per library; (**B**) boxplot of transformed data; (**C**) distribution of transformed data using a density plot; (**D**) PCA analyses indicate the substantial difference in thousands of genes induced by WBPH in *OxF3H* and wild plants, (**E**) Correlation matrix: results of the correlation analysis displayed in matrix format. Each row and each column represents a single sample. Colored cells indicate the correlation value between the row and column sample based on the read count for each gene. Green colors indicate lower correlation, and red colors indicate higher correlation; (**F**) statistics of DEGs (upregulated and downregulated) gene count comparisons of “*OxF3H*” vs. “Wild” at different times of infection for each comparison by using Deseq2. I:Genotype_trans-Times represent the number of genes whose expression was significantly affected by the interaction between genotype and time in *OxF3H* plants.

High-throughput sequencing resulted in 191.78 million high-quality, single-end reads (Tables S1–S3). An average of 89% of the clean reads were successfully aligned to the IRGSP 1.0 rice reference genome [18], and an average of 24,354 genes or protein-coding transcripts were identified between both genotypes and overall time points. This represents approximately 67.7% of the predicted transcripts in the rice reference genome IRGSP 1.0. Log2—transformation of the counts displays similar distributions between the samples (Figure 1).

Overall analysis of differentially upregulated and downregulated genes based on the experimental variables: (1) comparisons between the genotypes (“WT” and “*OxF3H*”) at each of the time points of infection; (2) genes that were differentially expressed over time of infection within a genotype; (3) genes that were differentially expressed based on an interaction between genotype and time of infection. Upregulation and downregulation were based on log 2-fold-change > 2 and log 2-fold-change < −2, respectively, for genes with adjusted *p*-value < 0.05.

The three comparisons between the experimental variables provide different types of information. Comparison (1), “WT” vs. “*OxF3H*” by the time of infection, gives a direct view of the differences in gene expression between the two genotypes at specific time points. The number of DEGs in the transgenic genotype (*OxF3H*) after 0 h and 3 h of infection were almost similar, averaging about 2000 genes. Notably, there was a much greater number of downregulated genes after 12 h and 24 h of infection. The highest number of DEGs between the two genotypes occurred after 24 h of infection, with over 4735 DEGs. This result indicates that significant differences in gene expression between the transgenic (*OxF3H*) and nontransgenic (“WT”) exist as the infection time increases (Figure 1F).

Similarly, comparison (2) indicates the effect of WBPH infection time on the expression level of genes within each genotype. Genes identified as DEGs would be any that have identified as DEGs with expression levels that significantly changed (up or down) over time. Overall, the differentially expressed genes were almost similar in “*OxF3H*” and “WT” after 3 h, 12 h, and 24 h of infection. However, in *OxF3H* plants, somewhat higher numbers of DEGs were found than in the WT. This does not indicate similar expression patterns over time, only that a similar number of genes exhibited significant changes in expression in different time pints.

Comparison (3) indicates the number of genes whose expression was significantly affected by the interaction between genotype and time. Genes identified as DEGs in this comparison are those whose expression was significantly different between the two genotypes in response to WBPH infection at different time points. These genes would be the most appropriate for further investigation as their expression differs between the “*OxF3H*” and “WT” genotypes over time.

The data depicted in Figure 1A can also be depicted by principal coordinate analysis (PCoA, Figure 1D) and a correlation matrix (Figure 1E). PCoA analysis of DEGs in “*OxF3H*” vs. “WT” was conducted using Qlucore software (v. 3.2). As illustrated in Figure 1D, gene expression in “*OxF3H*” and “WT” clustered more distantly at each time point in both genotypes. 

The correlation matrix (Figure 1E) provides a picture of the similarity of the samples, with the colour spectrum indicating how similar/dissimilar each sample is to another. The darker the red, the more directly similar the samples are, with the darkest red indicating a Pearson correlation of 1. Conversely, the green cells indicate samples that are very dissimilar. In general, the results confirm the results obtained in the PCoA. For example, 0 h (control) “WT” samples are similar to 0 h (control) “*OxF3H*” samples. Similar results were observed in after 3 h of infection in both genotypes. However, both 12 h and 24 h of infection exhibit different results in both genotypes. 

How “*OxF3H*” and “WT” compare in terms of upregulation and downregulation of gene expression is important to understanding the differences in response to WBPH infection in “*OxF3H*” relative to “WT”. A complex pattern of differential gene expression was evident, with numerous genes being upregulated or downregulated in *OxF3H* plants relative to “WT” plants (Figure 1F). The greatest disparity in the number of significantly upregulated and downregulated DEGs between “*OxF3H*” and “WT” occurred after 24 h, followed by 12 h. However, as compared to “WT”, greater number of DEGs were both up- and downregulated in “*OxF3H*” in all three time points (Figure 1F).

**Figure 2:**
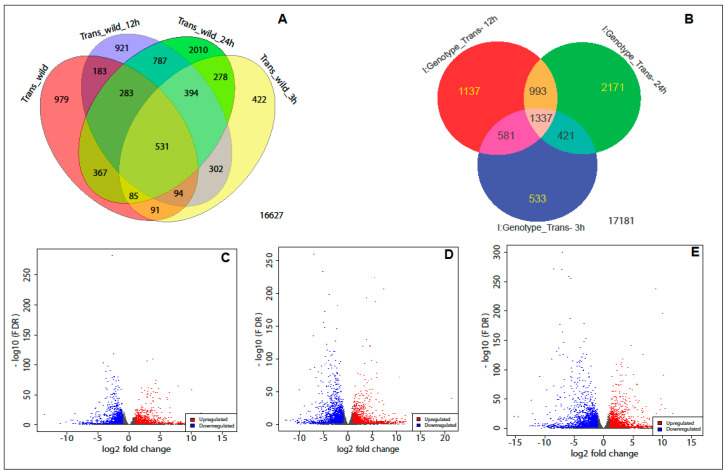
(**A**) Venn diagrams of the changes in differentially expressed genes in *OxF3H* (Trans) and “Wild” over time (0 h, 3 h, 12 h, and 24 h) of WBPH infection without interaction term; (**B**) Venn diagrams of the changes in differentially expressed genes only in *OxF3H* (Trans) over time (0 h, 3 h, 12 h, and 24 h) of WBPH infection; (**C**) volcano plot for differential expression analysis using DESeq2 after 3 h of WBPH infection in *OxF3H* plants; (**D**) volcano plot for differential expression analysis using DESeq2 after 12 h of WBPH infection in *OxF3H* plants; and (**E**) volcano plot for differential expression analysis using DESeq2 after 24 h of WBPH infection in *OxF3H* plants.

The Venn diagrams (Figure 2A,B; Table S4) of time to time differences in the number of unique DEGs within genotype indicate important differences in the gene expression level that may reflect differences in the response of both genotypes to WBPH. There were 531 DEGs common in control, 3 h, 12 h, and 24 h that were found both up- and downregulated in *OxF3H* compared to WT (Figure 2). Furthermore, in the interaction term between genotype and time, 1337 DEGs were found to be shared in the three time points (3 h, 12 h, and 24 h) after infection (Figure 2B). These DEGs were expressed only in *OxF3H* plants, not in WT plants. The highest DEGs 2171 were found differentially expressed only in *OxF3H* plants after 24 h of infection (Figure 2B). 

### 2.1. Gene Ontology (GO) Enrichment and Pathway Analysis

**Figure 3:**
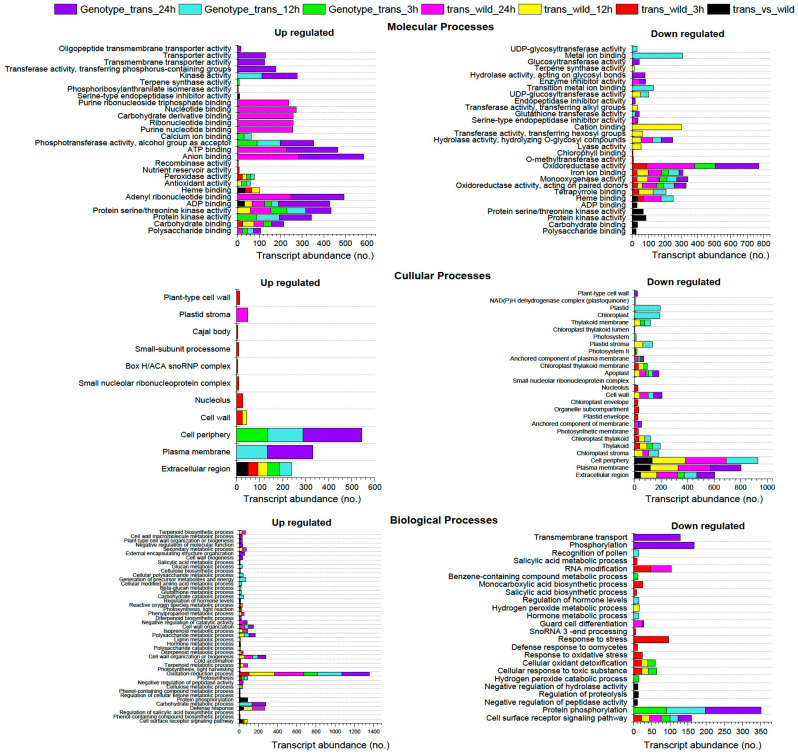
The abundance of transcripts of pathways under biological processes, cellular processes, and molecular processes in differential expression groups in *OxF3H* plants infested with WBPH at different time points. The different colors show different comparisons used in Deseq2 analysis.

DEGs were classified into three major GO categories: biological process, molecular function, and cellular component. Overall, 5427, 5847, and 11,494 GO terms were identified for biological process, cellular processes, and molecular processes, respectively, in both “*OxF3H*” and “WT” plants (Figure 3 and Figure S1). Among all comparisons, the highest number of biological GO terms was identified after 24 h in trans_wild_24 h and Genotype_trans_24 h comparison. Among these biological processes, the highest number of GO terms were related to oxidation-reduction processes (GO:0055114) in all comparisons. Similarly, in cellular processes, the highest GO terms were enriched related to cell periphery (GO:0071944), followed by plasma membrane (GO:0005886) and extracellular region (GO:0005576), in all comparisons (Figure 3; Table S5). Moreover, in molecular terms, the highest number of GO terms was enriched after 12 h of infection followed by 24 h of infection. Similarly, the highest GO term was related to oxidoreductase activity (GO:0016491) followed by monooxygenase activity (GO:0004497) in all comparisons. In pathway analysis using GAGE (generally applicable gene set enrichment) fold change values of all genes to identify coherently altered pathways, the results revealed that after early infection (3 h), pathways related to biological processes such as response to stress, salicylic acid metabolic process, defense response to oomycetes, cellular response to toxic substance, cellular oxidant detoxification, and other pathways were found to upregulated in the *OxF3H* genotype, while pathways related to terpenoid metabolic processes, photosynthesis, and oxidation-reduction process were found downregulated (Figure S1A–E). However, after 12 h of infection, regulation of hormone levels, hormone metabolic process, and cell surface receptor signaling pathways were upregulated (Figure 2A–E). Taken together, our data suggest that rice reprograms defense response, salicylic acid biosynthesis, and cellular oxidant detoxification to initiate the response to infection by WBPH.

### 2.2. DEGs Involved in the JA, BR, SA, and ET Signaling Pathways

**Figure 4:**
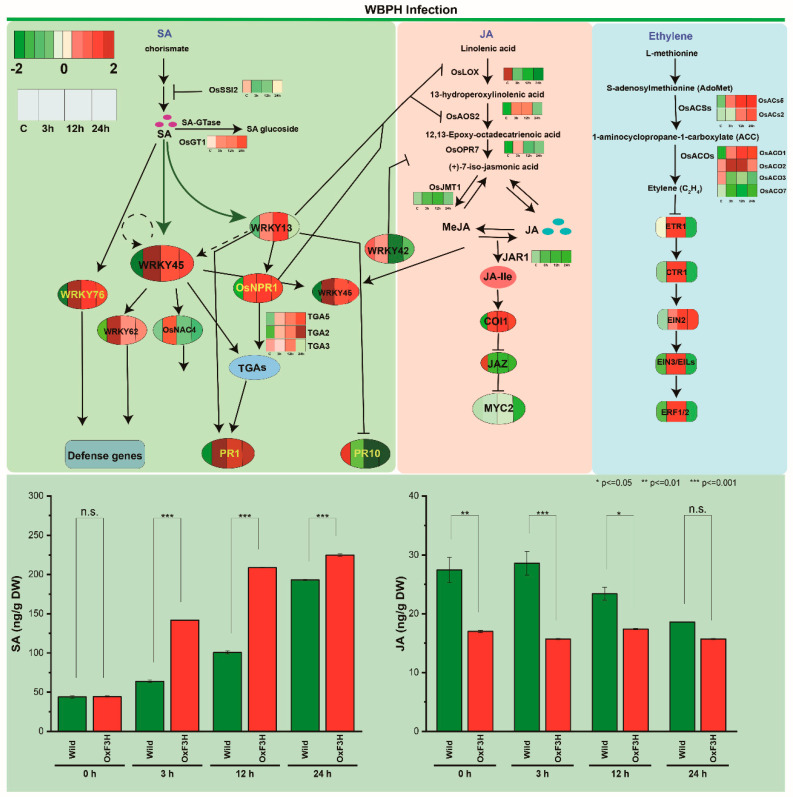
Transcriptional changes of salicylic acid, jasmonic acid, and ethylene biosynthesis pathway genes during WBPH infection in rice (*Oryza sativa*). Genes highlighted in red are upregulated, while those highlighted in green are downregulated in the RNA-seq dataset in *OxF3H* plants at different time points. JA and SA regulation in wild and transgenic plants during WBPH stress after 0 h, 3 h, 12 h, and 24 h. n.s. mean non significant.

To determine the defense phytohormones response in both “*OxF3H*” and “WT” plants against WBPH infestation were observed in all comparisons. The expression of genes involved in JA, ethylene (ET), salicylic acid (SA), and GA biosynthesis and signaling pathways is shown in (Figure 4). Induced expression of marker genes associated with the SA response were identified. These included the nonexpression of pathogenesis-related genes 1 *NPR1* (Os01g0194300), TGA transcription factors (Os01g0859500), and *NPR1*-dependent/independent pathogenesis-related (*PR*) gene (Os01g0382000). *NPR1* is a central positive regulator of SAR in Arabidopsis that transmits the SA signal to downstream *PR* gene activation [19]. SA accumulation triggered by pathogen infection alters cellular redox potential, resulting in the nuclear translocation of *NPR1* [20]. The SA pathway in rice branches into Os*WRKY*45- and *OsNPR1*-dependent pathways. *NPR1* then interacts with transcription factors (TFs) of the *TGA* family and activates target defense genes (Figure 4). The RNAseq data show that overexpression of *NPR1* (Os01g0194300), TGA transcription factors (Os01g0859500), and *NPR1*-dependent/independent pathogenesis-related (*PR*) gene (Os01g0382000) in *OxF3H* plants enhanced resistance to WBPH at different time points. Overall, four *TGA* transcriptional factors, such as *TGA2* (Os01g0808100), *TGA3* (Os03g0318600), and *TGA5* (Os01g0279900), were found to upregulated in *OxF3H* plants. Furthermore, other branches of SA pathways Os*WRKY*45 (Os05g0322900) expression were also significantly upregulated in the *OxF3H* plant. This overexpression confers very high resistance to WBPH. Overall, the SA biosynthetic pathway was found to be upregulated in *OxF3H* plants (Figure 4). Overexpression of *OsNPR1* in rice confers high levels of resistance to the leaf blight pathogen *Xanthomonas oryzae* pv. *oryzae* and the blast fungus *Magnaporthe oryzae,* which are associated with constitutive accumulation of pathogenesis-related (*PR*) transcripts [21,22].

**Figure 5:**
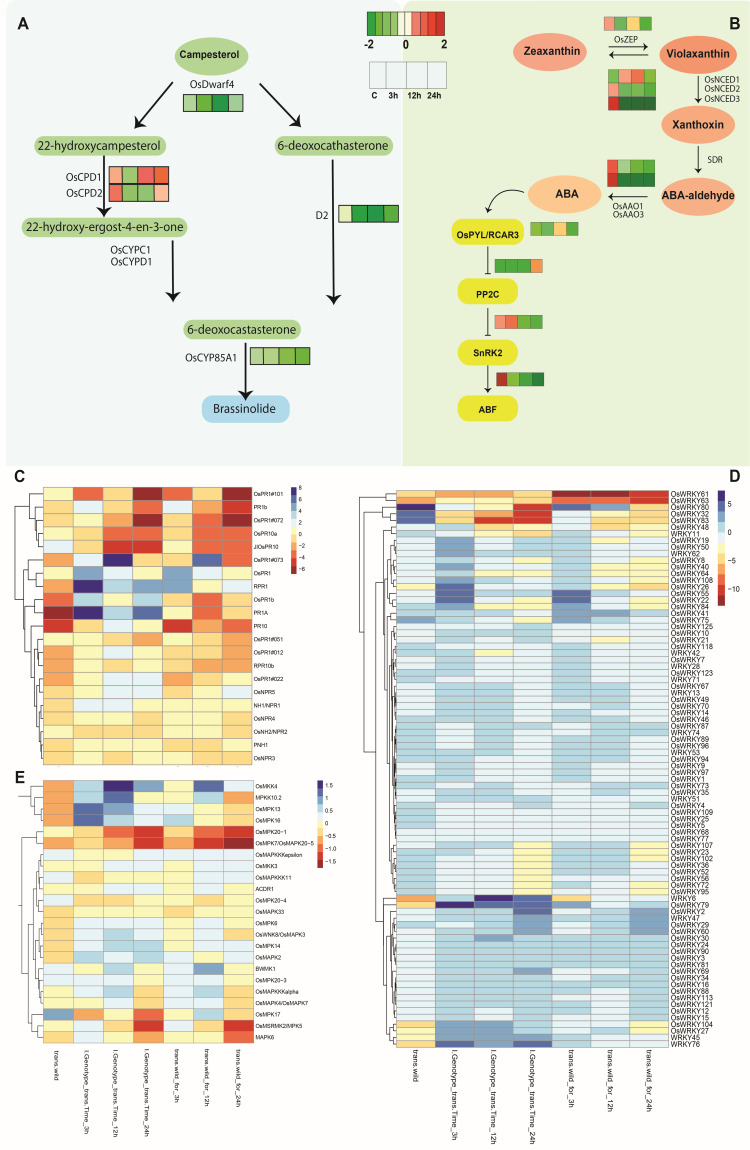
Transcriptional changes of abscisic acid and brassinosteroid biosynthesis pathway genes during white-backed planthopper infection in rice (*Oryza sativa*). (**A**) Abscisic acid biosynthesis and signaling genes; (**B**) brassinosteroid biosynthesis genes. Genes highlighted in red are upregulated, while those highlighted in green are downregulated in the RNA-seq dataset. (**C**) Heatmap shows the expression patterns of pathogenesis-related genes, (**D**) *WRKY* transcription factor, and (**E**) genes related to MAPK cascades.

The transcriptome data showed that the JA synthesis-related genes, lipoxygenases (*OsLOXs*), allene oxide synthase (*OsAOS*), and jasmonate O-methyltransferase (*OsJMT1*) were significantly downregulated in *OxF3H* plants following WBPH infection at all time points. Additionally, *OxF3H* plants exhibited upregulation of JA receptors (OsCOI) after infection but downregulation of the JA biosynthesis genes (*OsLOXs*, *OsAOS2*, *OsJMT1*). Similarly, the JA-responsive *PR* gene *JiPR10* was significantly downregulated at 3 h, 12 h, and 24 h of infestation (Figure 4). Likewise, the genes related to jasmonate ZIM-domain (*JAZ*) genes *OsJAZ9* (Os03g0180800), *OsJAZ3* (Os07g0153000), *OsJAZ11* (Os04g0395800), and *OsJAZ2* (Os03g0180900) were found downregulated in *OxF3H* plants. Like JA, expression of brassinosteroid-specific biosynthesis genes (*OsDWARF4*, *D2*, and *OsCPD*s) was downregulated during WBPH infection in *OxF3H* plants at all three time points. These results show that both JA and BR biosynthetic pathways were downregulated in response to WBPH infection in *OxF3H* plants (Figure 5A).

To validate the results of transcriptome sequencing, we investigated the accumulation of SA and JA in the “WT”, and “*OxF3H*” plants after 0 h, 3 h, 12 h, and 24 h after infestation by using the gas chromatography–mass spectrometry (GC-MS) technique. The results revealed a similar pattern, with transcriptomic data and the accumulation of SA consistently and significantly (*p* ≤ 0.001) increased in *OxF3H* plants compared to the WT (Figure 4). However, in the case of JA, the accumulation of JA was significantly reduced in the *OxF3H* and WT plants. The *OxF3H* plants showed more reduction than the WT plants at all time points. These results are similar to the previously reported results of Jan et al. [11], in which SA and JA regulate antagonistically in *O. sativa* during biotic stress (Figure 4). These results were consistent with the selected gene expression, as SA pathway related genes were upregulated in transgenic plants, and *JAZ2* genes related to the JA pathway were downregulated in transgenic plants. Cross-communication between the initiated hormone signaling pathways contributes to the activation of attacker-specific defenses [23,24,25,26]; (Figure 1). JA and SA are dominant players in the regulation of the immune signaling network, in which JA is generally effective against necrotrophic pathogens and insects, while SA is primarily effective against biotrophic pathogens [27]. Accumulating evidence indicates that ET can interact both positively and negatively with SA, depending on the plant attacker interaction [28]. For example, ET acts positively on the level of SA-mediated resistance against the pathogen *Leptosphaeria maculans* in *Brassica napus* [29], while it acts negatively on SA-mediated defense against the pathogen *P. syringae* in Arabidopsis [30]. Most studies on the modulating role of ET report on its effect on the JA signaling pathway and the interplay between the JA and SA pathways. Therefore, we focus on these ET-hormone interactions in more detail below.

Ethylene is another key regulator of plant development and defense. The hormone is elicited by insect attack and has been suggested to play an important role in induced resistance to herbivores [31,32,33]. Ethylene positively regulates the accumulation of defensive proteins and secondary metabolites, including phenolics, alkaloids, and terpenoids, as well as other volatile organic compounds [34,35,36,37], most probably by synergizing jasmonate (JA) signaling [32,35]. Therefore, in our transcriptomics data, we investigated changes in the expression of genes involved in ET biosynthesis in *OxF3H* plants that had been fed upon by the WBPH. In rice, a large gene family composed of different 1-aminocyclopropane-1-carboxylic acid (ACC) synthase genes (designated *OsACSs*) and ACC oxidase genes (designated *OsACOs*) controls ET biosynthesis [38,39]. The data revealed that the expression levels of most of the ET biosynthesis genes were upregulated after WBPH infestation, except *OsACS7* (Os07g0280200), which was found dowregulated at all three time points in *OxF3H* plants. Similarly, expression levels of three members of the *OsACS5* (Os01g0192900) were found to be highly upregulated at all time points compared to other genes in *OxF3H* plants. However, compared to *OxF3H* plants, all the *OsACS5* (Os01g0192900) genes were upregulated in infected *OxF3H* at the time of infection. Similar results were observed in a previous study, in which *OsACS* genes had increased expression levels after BPH infestation [40]. In addition, expression levels of four members of the ACC oxidase subfamily *OsACO1* (Os09g0451000) and *OsACO2* (Os09g0451400) were increased after 3 h, 12 h, and 24 h of infection, but then they began to decrease and reached their lowest levels after 24 h of WBPH feeding. Similar results were reported previously in rice [40]. The *OsACO3* (Os02g0771600) and *OsACO7* (Os01g0580500) expressions were downregulated at all time points. However, *OsACO5* (Os05g0149400) showed different results, and in the first two time points after 2 h and 12 h, it showed upregulation. After 24 h, however, it was completely downregulated in *OxF3H* plants.

Once ethylene is produced, it is perceived by its receptors. Several ethylene signaling components have been characterized, including *OsERS1*, *OsETR2*, *OsCTRs*, *OsEIN2*, and *OsEIL1/2* [41]. Ethylene-insensitive 2 (*EIN2*) is a membrane protein that acts as the central transducer of ethylene signaling pathways. *EIN3* and *EIN3-LIKE 1* (*EIL1*), two downstream components of *EIN2*, function as transcription factors to regulate the transcription of hundreds of genes responsive to ethylene [41]. *OsEIN2* and *OsEIL1/2* are positive regulators of ethylene signaling in rice, as mutation of *OsEIN2* or *OsEIL1/2* in rice results in complete ethylene insensitivity [42,43]. In our results, *OsETR* (Os02g0820900) and *OsCTR1* (Os02g0527600) genes were highly upregulated after 3 h and 12 h of infection in *OxF3H* plants. Similarly, the ethylene-insensitive 2 (*EIN2*) (Os07g0155600) is a membrane protein that acts as the central transducer of ethylene signaling pathways. *EIN3* (Os02g0200900) and *EIN3-LIKE 1* (*EIL1*) (Os03g0324300), two downstream components of *EIN2* (Os07g0155600), showed the same results and upregulated after 3 h and 12 h of infection. Furthermore, downstream in the ET signaling cascade, transcription factors of the ethylene response factor (*ERF*) family play a dominant role in regulating defenses. For example, *ERF3* was identified in rice (*Oryza sativa*) as a gene that positively affects the expression of trypsin proteinase inhibitors andtrypsin proteinase inhibitors’ expression and mediates resistance toward *Chilo suppressalis* caterpillars [44].

Upregulation of ERF transcriptional factor may be involved in rice (*OxF3H*) to enhance resistance to WBPH. In our analysis, OsERF (Os03g0860100) was found to be upregulated in *OxF3H* plants at the first two time points, while its expression decrease was upregulated in *OxF3H* plants at the first two time points, while its expression decreased at 24 h of infection. Previously, ERF1 [45] and octadecanoid-responsive Arabidopsis epetala2/ethylene response factor domain protein 59(ORA59) [46] were two *Arabidopsis* ERF transcription factors with roles in ET-dependent defense. Overexpressing or silencing these ERF transcription factors in Arabidopsis leads to enhanced or reduced resistance to several fungi [47]. Overall, *ERFs* are conserved among different plant species [48], as is mediate resistance to especially necrotrophic pathogens [49,50,51]. 

Although SA, JA, and ET are the three hormones most closely linked to plant defense, another important component is the stress-responsive hormone abscisic acid (ABA). Like other plant hormones, ABA also plays a dual role during biotic stress in plants. In most cases, however, ABA acts as a negative regulator of disease resistance with inhibition of ABA biosynthesis and/or signal transduction commonly resulting in enhanced disease resistance to a wide variety of bacterial, fungal, and oomycete pathogens exhibiting distinct parasitic habits [52,53,54,55,56,57,58,59]. In our transcriptome analysis, the expression of ABA biosynthetic genes *OsNCED2* (Os12g0435200) and *OsNCED3* (Os07g0154100) was found to be downregulated in *OxF3H* plants, and its downregulation steadily increased with infection time. Interestingly, transcription of the ABA-responsive genes *OsLip9* and *OsRab16* mirrored the profiles observed for *OsNCED2* and *OsNCED3*, strongly downregulated at 3 h, 12 h, and 24 h of infection (Figure 5). Moreover, we further analyzed the receptor complex *PYR*/*PYL*/*RCAR*, phosphatase type 2 Cs (*PP2Cs*), and *SNF-1*-related kinase (*SnRK2s*) genes that are positive regulators of ABA signaling (Figure 5B). *OsPYL* (Os02g0255500) and *SnRK2* (Os01g0869900) show significant overexpression after 12 h of infection. However, at 3 h and 24 h, they show downregulations. On the other hand, *OsPP2C08* (Os01g0656200) was found to be downregulated at all time points. The ABA-responsive elements (ABREs) that are recognized by transcription factors referred to more specifically as ABRE-binding proteins (AREBs) or ABRE binding factors (ABFs) were found to be downregulated in *OxF3H* plants during WBPH infection. The overall results show that ABA biosynthetic pathways in *OxF3H* were downregulated during WBPH infection.

Compared to the wealth of gathered knowledge regarding the regulatory role of GA in plant growth and development, relatively little attention has been paid to elucidate the involvement of GA and its signaling components in defense responses against plant pathogens [60,61]. However, evidence is accumulating on the crucial role of GA in plant responses to pathogen attack [60,61]. These studies suggest that GA plays a different role in plant immunity depending on the host and the pathogen involved [62]. In our study, enzymes involved in GA biosynthesis: ent-copalyl diphosphate synthase (*OsCPS1*) (Os02g0278700), ent-kaurene synthase (*KS*) (OS04G0612000), and ent-kaurene oxidase (KO) (Os06g0569500) were found to be downregulated in *OxF3H* plant after infection. Similarly, GA-gibberellin-insensitive dwarf 1 (*GID1*) (Os03g0790500) and DELLA protein *SLR1* (Os01g0646300) were downregulated in *OxF3H* plants during WBPH infection. Here, SA interferes with GA signaling and stabilizes the DELLA protein SLR1, resulting in the amplification of SA-dependent defense responses. 

### 2.3. Expression of Pathogenesis-Related Expression (PR) and Nonexpressor (NPR) Genes

We investigated the expression of PR genes and their roles in rice defense response to WBPH in both “*OxF3H*” and “WT” using three time points. The comparison (3) interaction term indicates that thirteen PR genes were found both upregulated and downreulated in both plants (*OxF3H* and WT) for different lengths of infection. From the comparison *OsPR1b* (Os01g0382000), *PR1A* (Os07g0129200), *OsPR1* (Os07g0129300), and *OsPR1*#012 (Os01g0382400) were found to be significantly upregulated in *OxF3H* plants after 3 h, 12 h, and 24 h of infection (Figure 5C). However, *OsPR10a* (Os12g0555500), *OsPR1*#101 (Os10g0191300), and *JIOsPR10* (Os03g0300400) were found to be downregulated at all three time points in *OxF3H* plants. Among these genes, OsPR1b (Os01g0382000) and *PR1A* (Os07g0129200) were significantly induced in the *OxF3H* plants after 3 h and 24 h of infection. Interestingly, all these genes were found to be to downregulated at 0 h, before infection, in *OxF3H* plants. Moreover, we have analyzed the expression pattern of the *NPRs* (NHs) gene in *OxF3H* plants and found that nonexpression of pathogenesis-related gene 1, *NPR1* (also known as *NIM1* and *SAI1*), is a key regulator of SA-mediated systemic acquired resistance (SAR) in Arabidopsis. This gene was significantly upregulated in *OxF3H,* and the highest fold change was observed after 3 h, followed by 12 h. Among five *NPR1* homologs in rice, four—*NPR1* (Os01g0194300), *OsNPR3* (Os03g0667100), *OsNPR4* (Os01g0837000), and *OsNPR5* (Os01g0948900)—were found upregulated in *OxF3H,* while only *OsNH2* (OS01G0767900) was found to be downregulated. 

*NPR1* is involved in multiple plant defense responses, including the jasmonic-acid-mediated responses to chewing insects [63] and induced systemic resistance triggered by rhizobacteria [64]. However, one likely central function of *NPR1* is the regulation of defense gene expression through its interactions with *TGA* factors [65]. *TGA* factors are likely to have distinct roles in mediating gene expression. Therefore, we have observed *TGA* factor genes in both genotypes. Our results revealed that three *TGA* genes, *TGA2* (Os01g0808100), *TGA3* (Os03g0318600), and *TGA5* (Os01g0279900), were found to be significantly upregulated in *OxF3H* plants. Together, these data suggest that the three *TGA* genes may be involved in *OxF3H* defense gene regulation though interaction with the *NPR1* rice homolog.

### 2.4. WRKY Transcription Factor and MAPK Genes Expression during WBPH Stress

**Figure 6:**
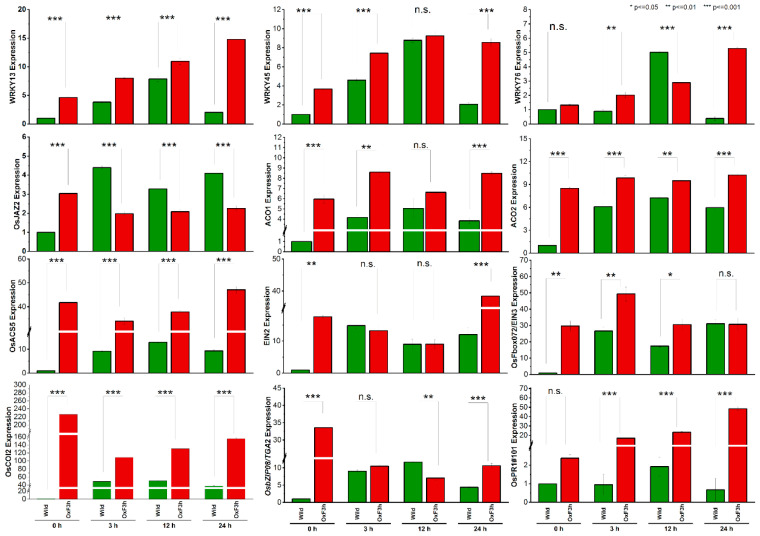
Validation of relative expression of selected genes in wild type and *OxF3H* plants under WBPH infection, by qRT-PCR. The fold change of each gene was calculated at different time points (0 h, 3 h, 12 h, and 24 h). Graphs show mean ± standard deviation of three biological replicates, and asterisks show significant differences (* *p* ≤ 0.05, ** *p* ≤ 0.01, and *** *p* ≤ 0.001) according to ANOVA and Bonferroni post hoc tests. n.s. mean non significant.

We investigated the expression of *WRKY* transcriptional factor genes and their roles in rice defense response to WBPH in both “*OxF3H*” and “WT” using three time points. These results indicate that about 30 *WRKY* genes were significantly expressed in comparison (3) after 3 h, 6 h, and 24 h of infection in *OxF3H* plants. Various important *WRKY* transcription factor genes, such as *WRKY*76, *WRKY*79, *WRKY*6, *WRKY*45, *WRKY*26, *WRKY*19, *WRKY*50, *WRKY*55, and *WRKY*104, were found to be more upregulated in *OxF3H* plants than in WT plants after WBPH infection (Figure 5D). Previous reports suggested that Os*WRKY*6 appears to play a role in defense signaling in rice by activating the expression of the reporter gene located downstream of *OsPR1* promoter, further demonstrating that a defense-associated gene, *OsPR1*, is a target gene of Os*WRKY*6 [66]. Similarly, Os*WRKY*45 and Os*WRKY*76 were found to confer rice resistance to *Mangaporthe oryzae* but reduced resistance to the bacterial pathogens Xoo and *Xanthomonas oryzae* pv *oryzicola* (Xoc) and to cold and drought stresses [67,68,69]. Interestingly, *WRKY*22, *WRKY*84, *WRKY*107, *WRKY*23, *WRKY*8, *WRKY*7, *WRKY*28, *WRKY*40, and *WRKY*64 were upregulated in “*OxF3H*” only after 3 h of infection. Similar upregulation of the above *OsWRKY* genes was observed in “WT” to a lesser extent than in *OxF3H* plants. Other important WRKY genes, *WRKY*32, *WRKY*80, and *WRKY*83, were significantly expressed (downregulated) in *OxF3H* plants during WBPH infection after 3 h, 12 h, and 24 h of infection (Figure 5D). However, these genes were found tob e upregulated in “WT” plants after 3 h of infection, while their expressions were decreased after 12 h and 24 h of infection. It is noteworthy that these genes were found to be highly upregulated in *OxF3H* plants in control conditions (Figure 6). Two *WRKY* genes, *WRKY*61 and *WRKY*63, were found to be highly downreggulated in “*OxF3H*” plants, while their expressions became normal in interaction terms only in *OxF3H* plants.

MAPK cascades also have roles in resistance to herbivores. However, a number of rice MAPK genes show diverse transcriptional patterns upon herbivores infection [70]. Our transcriptome analysis revealed that *OsMKK4* (Os03g0843000), *MPKK10.2* (Os03g0225100), *OsMPK13* (Os02g0135200), and *OsMPK16* (Os11g0271100) were upregulated in *OxF3H* plants during WBPH infestation at 3 h, 12 h, and 24 h. However, the expression levels of the first two were highly upregulated after 12 h, while *OsMPK13* and *OsMPK16* were highly expressed after 3 h of infection. These genes were found to be upregulated in WT plants, but their expressions were lower than in *OxF3H* plants (Figure 5E). On the other hand, *MAPK6* was found to be upregulated in *OxF3H* plants after 3 h and 12 h of infection, it downregulated after 24 h of infection. Recently, two *MAPKs*, *OsMPK4* and *OsMPK6* (*OsMPK1*), phosphorylated *OsWRKY45* protein in vitro, and the activity of *OsMPK6* (*OsMPK1*) was rapidly upregulated by SA treatment in rice cells [71]. Similarly, the *OsMPK20* gene was upregulated in F3H plants after 3 h of infection, while in WT plants, it was downregulated. The present results suggest that rice *MPKK10.2* positively regulates WBPH resistance in *OxF3H* plants, as reported previously in rice in bacterial resistance and drought tolerance via phosphorylating and activating two distinct *MAPKs*, *MPK6* and *MPK3* [72]. MAP kinase signaling has been reported to be involved in both pathogen-associated molecular-pattern-triggered immunity (PTI), or basal resistance, and effector-triggered immunity (*ETI*), or race-specific resistance [73]. Brown planthoppers (BPHs) mediated enhancements in transcriptional levels of OsMPK20-5, negatively regulating the rice resistance to BPH by modulating ethylene and nitric oxide accumulation [74]. Though the role of *MAPKs* in herbivore-induced plant defense has been examined extensively, we face a paucity of data with respect to the function of *MKKs* in defense against herbivores.

### 2.5. RNA-Seq Data Validation by RT-qPCR

Different genes were randomly selected from all the three (SA, JA, ET) pathways to validate the RNA-seq data assayed by RT-qPCR (Figure 6). We compared the wild type group to transgenic group according to their respective time points. A total of six genes, namely *WRKY13*, *WRKY45*, *WRKY76*, *TGA2*, *PR1,* and *PR10,* were selected related to the SA pathway and assayed via RT-qPCR. *COI2* and *JAZ2* were selected related to the JA pathway, and five genes—ACO1, ACO2, ACS5, EIN2, and EIN3—were selected related to the ethylene pathway. The relative expression changes in RT-qPCR results were highly consistent with the RNA-Seq data. All the selected genes were upregulated in transgenic plants, only *PR10* (SA pathway) and *JAZ2* (JA pathway) were downregulated in transgenic plants as compared to wild type plants.

## 3. Materials and Methods

### 3.1. Plant Material

Nontransgenic “WT” and transgenic “*OxF3H*” nagdong rice seedlings that were approximately 21 days old were used in this study. *OxF3H* was initially described in our previous article [11]. *OxF3H* and wild-type plant seeds were sterilized with fungicides overnight, washed three times, and incubated for 3 days at 30 °C. Approximately 20 germinated seeds of each (wild type and *OxF3H*) were transferred to autoclaved soil contained in individual pots in multiples of three and grown in a growth chamber for one week. Seedlings were transferred to a greenhouse, and about 200 male and female WBPH (*Sogetella furcifera* (Horvath, 1899), Homoptera: Delphacidae) (2nd and 3rd instar) were separately introduced to both *OxF3H* and wild-type plants after three weeks of growth, at a ratio of 3 WBPH per plant. We used the same ratio of pests in our previous study [11], and the gene transcriptional level was significantly regulated, as shown in Supplementary Figure S2. The plants were kept in growth chamber as well as in green house at 28/26 °C during the 16/8 h light/dark photoperiod. The plants were treated with WBPH in the insectarium, which had 50 cm × 50 cm × 40 cm length, width, and height, respectively. Before introductions, WBPH were kept in a beaker with wet tissue for 2 h to starve them. The samples for RNA isolation were collected after 0 h (before WBPH infestation at 12 pm), 3 h, 12 h, and 24 h of WBPH inoculation. The 0 h time point of wild plants was considered the control. The RNA was isolated from the infected plants, and we randomly selected those plants that were attacked by most of the WBPH, as shown in Supplementary Figure S3. The WBPH were provided by the National Institute of Crop Science, Rural Development Administration, Korea. 

### 3.2. RNA Extraction, Library Construction, and Sequencing

RNA was isolated from fresh leaves of WT and *OxF3H* plants after 0 h, 3 h, 12 h, and 24 h of WBPH inoculation as, with purification and library construction as previously described by Liao et al. [75]. In brief, 1 mL Sarkosyl of 20% (*w*/*v*) was added to 10 mL of extraction buffer (2% CTAB, 2% polyvinylpyrrolidone (PVP) K-30 (soluble), 100 mM Tris HCl (pH 8.0), 25 mM EDTA, 2.0 M NaCl, 0.5 g/L spermidine (free acid) (HS), and 2% β-mercaptoethanol (added just before use). The extracted total RNA was dissolved in EB buffer (Qiagen, Germantown, MD, USA) supplemented by 1× Ambion RNA secure (Invitrogen/Life Technologies, Carlsbad, CA, USA). To activate RNA secure, the samples were incubated at 60 °C (in a water bath) for 10 min and then immediately put on ice. RNA quantity and quality were evaluated using a Nano-drop 1000 (Thermo Scientific, Waltham, MA, USA). Immediately prior to mRNA isolation, RNA samples were treated with DNase I (amplification grade, Invitrogen) at 37 °C for 30 min followed by heat inactivation at 65 °C for 15 min. Each RNA sample was adjusted to contain 5 μg of total RNA. Library construction was performed and run in two lanes using the Illumina HiSeq2000 platform to obtain 51 bp single-end reads. Libraries from three independent biological replicates of each genotype at each time point were sequenced and analyzed.

### 3.3. Bioinformatics Analyses

The analyzed data comprised 16 datasets—8 for the “WT” wild-type genotype and 8 for the *OxF3H* transgenic genotype of nagdong rice cultivar. Each genotype was sampled once, and each sampling time point contained two independent samples at each time point. After the 16 datasets were sequenced, each was run through an optimized RNA-seq pipeline to determine statistically significant differences in gene expression between specific comparisons. The IRGSP 1.0 rice reference genome and annotation used for the assembly and annotation were downloaded from Ensemble Plants [18].

A computational pipeline of optimized tools was used to identify differences in gene expression between the WT and “*OxF3H*” genotypes. The pipeline consisted of: (1) read quality check using FastQC [76]; (2) data trimming using Trim Galore [77]; (3) reference genome indexing using HISAT2 [78]; (4) alignment of trimmed reads to indexed reference genome using HISAT; (5) read count quantification using Feature Count (subread_v2.0.2); and (6) differential expression analysis using [79] in R.

Three distinct comparisons of differential gene expression were considered: (1) pairwise comparisons of “WT” vs. *OxF3H* at each time point; (2) separate time main effect for each genotype (“WT” and *OxF3H*); (3) interaction effect of genotype and time. The three types of comparisons provided a total of 13 comparisons, with comparison 1 being responsible for four, comparison 2 being responsible for six, and comparison 3 being responsible for three. The three types of comparisons provided a comprehensive overview of the changes in gene expression corresponding to genotype (comparison 1), time differences (comparison 2), and which genes exhibited expression patterns that differed due to genotype over the course of the entire study (comparison 3).

The specific results for differential gene expression were determined using DESeq2, which implements a Wald test or likelihood ratio test to determine which genes exhibit different transcript levels within a respective comparison. The pairwise comparisons (1 and 2) utilize the Wald test approach, which performs a parametric significance test of the selected factor level using a negative binomial distribution. Significant *p*-values result from the factor being determined as significant in the Wald test. The more complex comparisons (3) utilize a likelihood ratio test, which compares a full linear model considering appropriate additive and interactive effects and compares the fit against a reduced linear model with the selected factor(s) removed. Significant *p*-values result from a significant fitted improvement in the full model over the reduced model.

DESeq2 compiles a results file containing the gene ID, mean expression value, log 2-fold-change and standard error, statistical test value, *p*-value, and adjusted *p*-value. DESeq2 adjusts the *p*-values to account for multiple testing using an FDR method. For this study, genes were considered differentially expressed if their adjusted *p*-values were below 0.05. Principal coordinates analysis (PCoA) was performed using Qlucore v3.2 (Qlucore, Lund, Sweden) bioinformatic software, with statistical significance set at 0.05.

### 3.4. Reverse Transcription Quantitative PCR (RT-qPCR)

RT-qPCR was conducted on selected genes to validate the results obtained by the RNA-Seq analysis, as per [80]. Total RNA was diluted to 100 ng/μL. RT-qPCR analysis was performed using the Invitrogen SuperScript III Platinum SYBR Green One-Step RT-qPCR (South Korea). For selected gene expression, standard cDNA was synthesized using qPCRBIO cDNA Synthesis Kits from PCRBIOSYSTEMS (South Korea), following the manufacturer’s instructions. For quantitative RT-PCR, we used the StepOnePlus Real-Time PCR System, Life Technologies Holdings Pte Ltd. (Singapore), BioFACT™ 2X Real-Time PCR Master Mix (Including SYBR^®^ Green I) to relatively quantify the expression levels of selected genes. The primers were designed using primer three program and are listed in (Table S6). The primers were validated via blasting in an NCBI. To standardize the level of expression of each gene, actin was used as a housekeeping gene in each reaction, and the expression level was calculated in wild plants infested with WBPH relative to *OxF3H* infested with WBPH. The reaction was performed in a 20 µL volume containing 7 µL ddH_2_O, 1 µL primer, 10 µL 2X Real-Time PCR Master Mix, and 1 µL cDNA, with each reaction repeated three times. Three technical replicates were used for each of three biological replicates.

### 3.5. Quantification of Endogenous SA and JA

For SA and JA analysis, fresh leaves were ground into powder in liquid nitrogen. About 0.3 g of leaf powder was mixed with 90% and 100% ethanol and methanol, respectively, and centrifuged for 20 min at 1000 rpm. The supernatant was collected and dried in the vacuum drier, and the dried sample was resuspended in 3 mL TCA (5%). The resuspended sample was further mixed with ethyl acetate/cyclopentane/isopropanol (49.5:49.5:1, *v*/*v*), and the upper layer was collected and dried with nitrogen gas. A sample of approximately 1 µL was injected into HPLC for SA quantification. For JA analysis, about 0.5 g fresh leaves was grounded in liquid nitrogen. About 0.2 g powder was homogenized with acetone and 50 mM citric acid (70:30, *v*/*v*). The suspension was added to the internal standard [9,10-2H2]-9,10-dihydro-JA (20 ng). The extract was kept overnight at low temperature to evaporate highly volatile organic solvents. The remaining extract was filtered and extracted three times with 10 mL diethyl ether. The extract was loaded onto a solid-phase extraction cartridge (500 mg of sorbent, aminopropyl) and the cartridges were cleaned with 7.0 mL 2-propanol and trichloromethane (1:2, *v*/*v*). JA and standards were eluted with 10 mL diethyl ether and acetic acid (98:2, *v*/*v*). The residue of solvents after evaporation was esterified with diazomethane and analyzed by injecting a 1 µL sample into a GC-MS (6890 N network GC system and the 5973-network mass-selective detector (Agilent Technologies, Palo Alto, CA, USA) in the selected ion mode. The ion fragment was monitored at *m*/*z* = 83 amu, consistent with the base peaks of JA and [9,10-2H2]-9,10-dihydro-JA; JA was quantified using the peak areas corresponding to the respective standards.

## 4. Conclusions

Conclusively, over the last 30 years, there has been an increased effort to utilize biotechnology for improving biotic stress tolerance in crop species. In this paper, we investigated the effects of overexpressing the *OsF3H* gene in *Oryza sativa* during WBPH infestation by using transcriptomic data analysis. Our findings suggest that rice modulates it defense responses, salicylic acid biosynthesis and cellular oxidant detoxification, to initiate the response to WBPH infestation. Similarly, *OxF3H* plants showed altered expression of genes involved in pathogen responses and genes involved in ABA, JA, SA, BR, and ET-related pathways and their interaction, which could contribute to tolerance in these plants. Such studies are not only important in that they dissect plant responses to WBPH infestation at different times of infestation, but information derived from this study is required to consciously combine relevant resistance strategies against WBPH for its effective management.

## Data Availability

All data supporting the findings of this study are available within the paper and within its Supplementary Materials.

## Supplementary Materials

The following supporting information can be downloaded at: https://www.mdpi.com/article/10.3390/ijms232315308/s1.
