# Supplementary material for: Enhancing the Expression of the OsF3H Gene in Oryza sativa Leads to the Regulation of Multiple Biosynthetic Pathways and Transcriptomic Changes That Influence Insect Resistance
Source: Int J Mol Sci. 2022 Dec 4;23(23):15308. doi: 10.3390/ijms232315308 (PMC9737463; doi:10.3390/ijms232315308)
Supplement: Supplementary file 1 [file ijms-23-15308-s001.zip › ijms-2073349-supplementary figures.pdf]

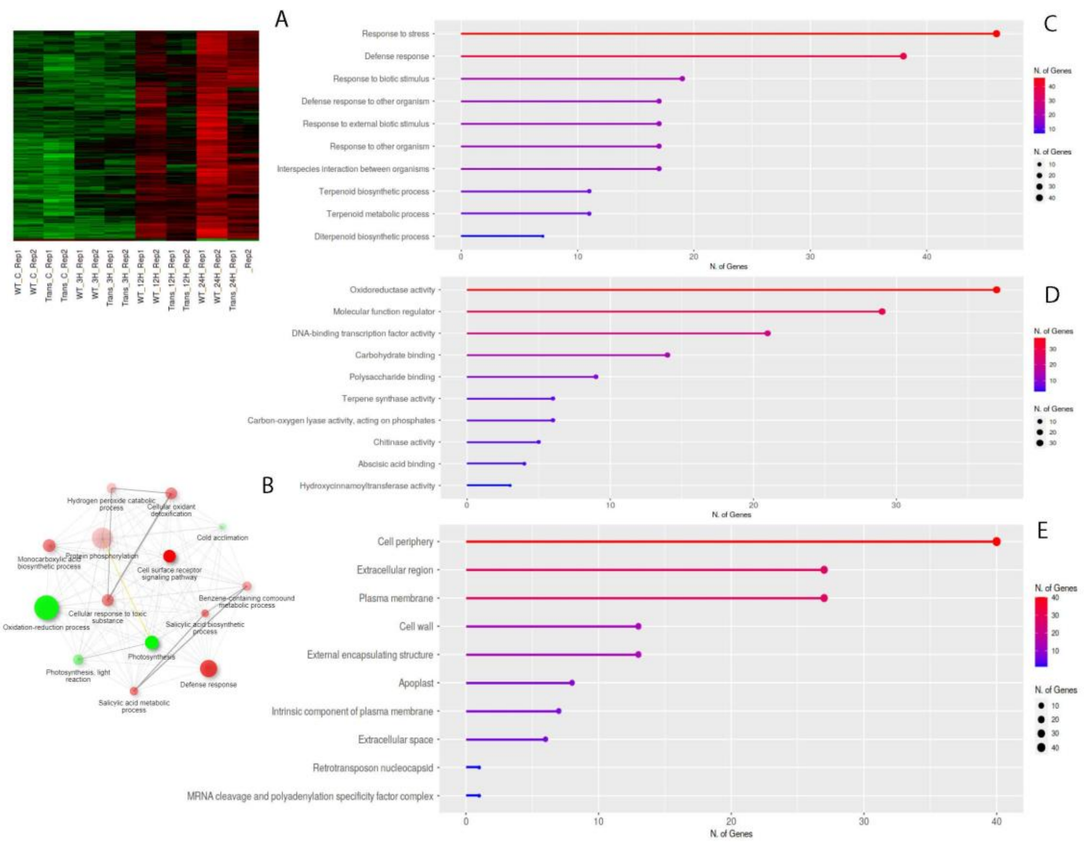

Figure S1. (A) Heatmap indicate the substantial difference in thousands of genes induced by WBPH in *OxF3H* plant at different time of infection, (B) Visualization of the differentially expressed genes using network analysis in *OxF3H* plants after WBPH infection, (C) Gene Ontology (GO) categories of the differentially expressed genes (DEGs) Biological process (D) Cellular components and (E) Molecular function.

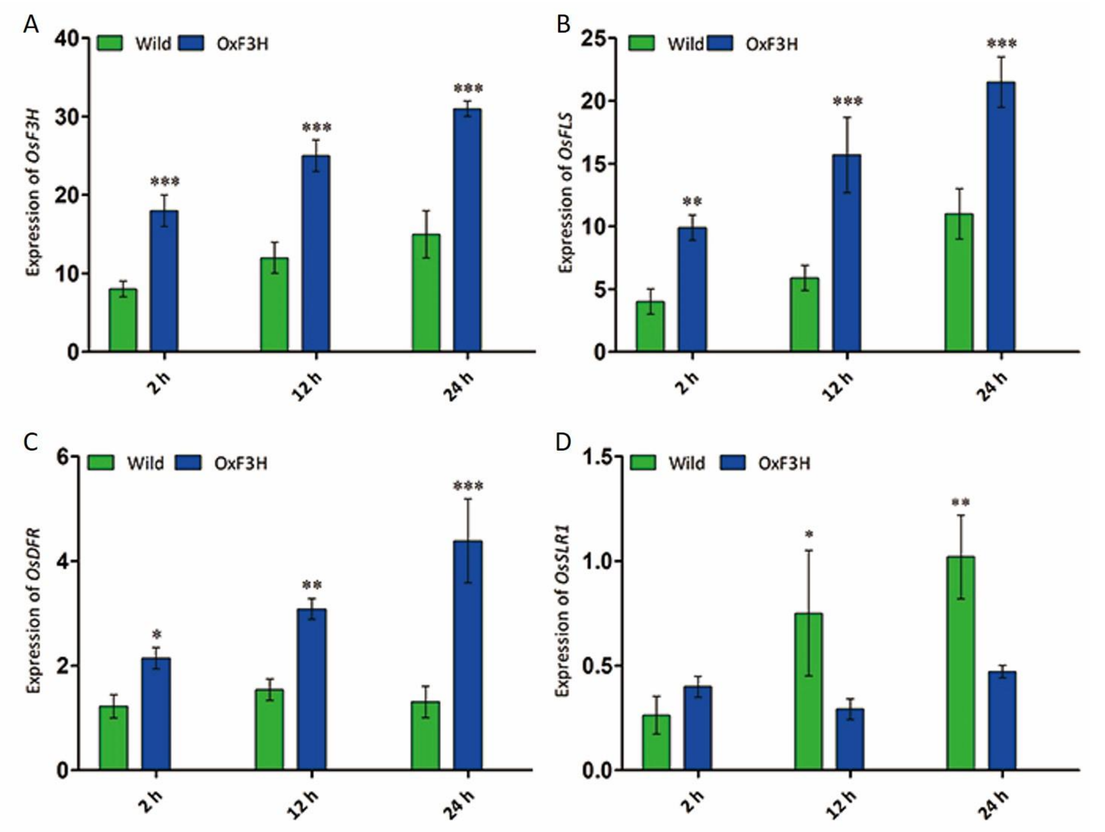

Figure S2. Gene expression data. (**A**) *OsF3H*, (**B**) *OsFLS*, (**C**) *OsDFR*, (**D**) *OsSLR1*. The fold change of each gene was calculated at different time points (2 h, 12 h, and 24 h). Graphs show mean±standard deviation of three biological replicates, and asterisks show significant differences (*p $\leqslant$ 0.05, **p $\leqslant$ 0.01, and ***p $\leqslant$ 0.001) according to ANOVA and Bonferroni post hoc tests. (published data).

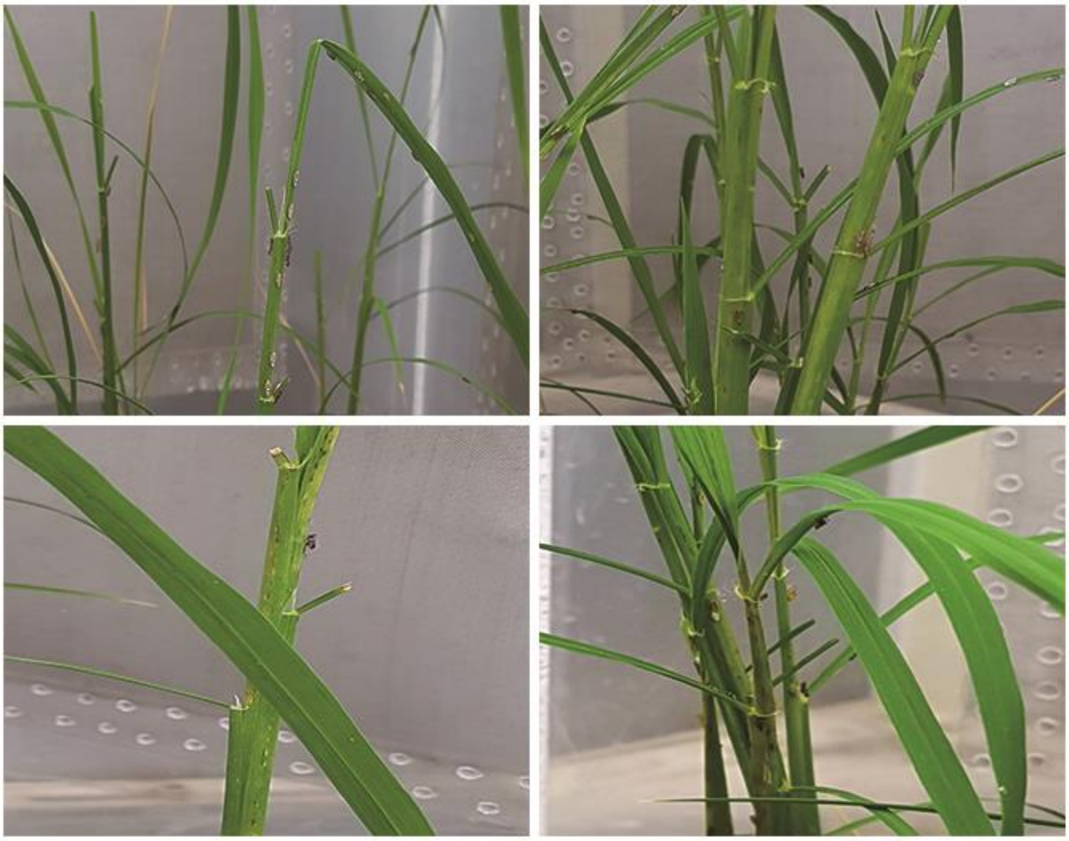

Figure S3. WBPH infected plants at different time intervals. We collected the samples fro RNA isolation from those plants that were attacked by most of the WBPH.
