# Supplementary material for: Enhancing the Expression of the OsF3H Gene in Oryza sativa Leads to the Regulation of Multiple Biosynthetic Pathways and Transcriptomic Changes That Influence Insect Resistance
Source: Int J Mol Sci. 2022 Dec 4;23(23):15308. doi: 10.3390/ijms232315308 (PMC9737463; doi:10.3390/ijms232315308)
Supplement: Supplementary file 1 [file ijms-23-15308-s001.zip › Supplementary tables.pdf]

Supplementary tables

Table S1. Raw data read information

Sample Name	Read Length (bp)	Total Base (bp)	Read Count	GC (%)	Q20 Ratio (%)	Q30 Ratio (%)
WT_C_Rep1	151	5,489,916,211	36,357,061	54	99.89%	97.50%
WT_C_Rep2	151	4,115,572,648	27,255,448	54.5	99.91%	96.80%
Trans_C_Rep1	151	5,010,381,132	33,181,332	53.5	99.88%	97.50%
Trans_C_Rep2	151	5,420,212,648	35,895,448	54.5	99.93%	97.40%
WT_3H_Rep1	151	5,905,519,249	39,109,399	55.5	99.82%	97.10%
WT_3H_Rep2	151	5,441,207,235	36,034,485	56.5	99.92%	97.30%
Trans_3H_Rep1	151	4,764,860,568	31,555,368	55	99.92%	96.80%
Trans_3H_Rep2	151	5,395,248,573	35,730,123	54.5	99.81%	97.10%
WT_12H_Rep1	151	6,464,777,345	42,813,095	54.5	99.83%	97.20%
WT_12H_Rep2	151	4,577,771,870	30,316,370	55	99.91%	97.20%
Trans_12H_Rep1	151	4,466,430,510	29,579,010	54.5	99.92%	97.10%
Trans_12H_Rep2	151	5,285,092,261	35,000,611	54.5	99.86%	97.30%
WT_24H_Rep1	151	4,221,921,797	27,959,747	53	99.93%	97.20%
WT_24H_Rep2	151	5,325,365,622	35,267,322	52.5	99.84%	97.20%
Trans_24H_Rep1	151	4,227,359,156	27,995,756	54.5	99.92%	97.20%
Trans_24H_Rep2	151	4,654,043,329	30,821,479	53.5	99.86%	97.00%

Table S2. Read information after Trimming with Trimmomatic

Sample Name	Total reads	Both Surviving	Forward Only Surviving	Reverse Only Surviving	Drop
WT_C_Rep1	36357061	36,002,720	257,820	47,396	49,125
		99.03%	0.71%	0.13%	0.14%
WT_C_Rep2	27255448	26,347,161	712,313	61,925	134,049
		96.67%	2.61%	0.23%	0.49%
Trans_C_Rep1	33181332	32,330,427	599,146	89,424	162,335
		97.44%	1.81%	0.27%	0.49%
Trans_C_Rep2	35895448	33,384,159	1,852,171	117,488	541,630
		93.00%	5.16%	0.33%	1.51%
WT_3H_Rep1	39109399	38,116,498	677,307	132,710	182,884
		97.46%	1.73%	0.34%	0.47%
WT_3H_Rep2	36034485	33,328,147	1,943,240	161,823	601,275
		92.49%	5.39%	0.45%	1.67%
Trans_3H_Rep1	31555368	29,104,073	1,783,664	127,644	539,987
		92.23%	5.65%	0.40%	1.71%
Trans_3H_Rep2	35730123	34,513,906	806,366	185,380	224,471
		96.60%	2.26%	0.52%	0.63%
WT_12H_Rep1	42813095	41,158,032	1,177,016	155,260	322,787
		96.13%	2.75%	0.36%	0.75%
WT_12H_Rep2	30316370	27,965,407	1,674,443	161,693	514,827
		92.25%	5.52%	0.53%	1.70%
Trans_12H_Rep1	29579010	27,442,236	1,587,781	80,321	468,672
		92.78%	5.37%	0.27%	1.58%
Trans_12H_Rep2	35000611	33,933,335	731,474	125,963	209,839
		96.95%	2.09%	0.36%	0.60%
WT_24H_Rep1	27959747	26,823,148	933,384	19,116	184,099
		95.93%	3.34%	0.07%	0.66%
WT_24H_Rep2	35267322	34,746,592	394,752	54,795	71,183
		98.52%	1.12%	0.16%	0.20%
Trans_24H_Rep1	27995756	26,563,345	1,162,340	25,509	244,562
		94.88%	4.15%	0.09%	0.87%
Trans_24H_Rep2	30821479	30,344,529	362,326	40,243	74,381
		98.45%	1.18%	0.13%	0.24%

Table S3. Read Alignment results with Hisat2

Sample Name	Overall Alignment Rate	Concordant Zero	Concordant Pair Alignment	Multiple Alignment
WT_C_Rep1	97.67%	4.17%	83.64%	12.19%
WT_C_Rep2	97.09%	5.56%	89.82%	4.62%
Trans_C_Rep1	98.19%	3.75%	87.34%	8.91%
Trans_C_Rep2	98.13%	4.74%	90.78%	4.49%
WT_3H_Rep1	98.50%	3.18%	79.43%	17.39%
WT_3H_Rep2	98.44%	4.19%	91.13%	4.68%
Trans_3H_Rep1	97.70%	5.38%	89.88%	4.74%
Trans_3H_Rep2	97.97%	3.91%	76.61%	19.47%
WT_12H_Rep1	95.02%	6.82%	79.09%	14.09%
WT_12H_Rep2	95.08%	7.55%	87.69%	4.76%
Trans_12H_Rep1	95.79%	7.04%	87.64%	5.32%
Trans_12H_Rep2	96.47%	5.32%	79.11%	15.58%
WT_24H_Rep1	84.77%	17.22%	78.79%	3.99%
WT_24H_Rep2	83.87%	17.47%	67.26%	15.27%
Trans_24H_Rep1	97.75%	4.99%	90.80%	4.21%
Trans_24H_Rep2	97.80%	4.02%	79.96%	16.02%

Tables S4 and S5 are in excel file.

Table S6: Primers used for qRT-PCR.

Gene name	Forward primer	Reverse Primer	Amplicon length
WRKY13	ATGATCAGTATGGCGTGTCG (Sense)	CCGAGCACCGGTAGTAGTTC (Antisense)	173
WRKY45	GGGAATTCGGTGGTCGTCAA (Sense)	TTTGGATCTCCTTCTGCCCG (Antisense)	73
WRKY76	AGCTGCCCGAATTCTAGCTT (Sense)	AGGATCGTGAGGCCCGATAG (Antisense)	242
OsJAZ2	AGGTTTCGTCCTCGTTTCGT (Sense)	TACCGGCCATGTTGTAGCTC (Antisense)	103
ACO1	ACTCCATCGTCGTCAACCTC (Sense)	GAACACGAACTTGGGGTACG (Antisense)	209
ACO2	TCGACATGAGTCTGCTCGAC (Sense)	ACGCTTGTAGTGGTCCTTGG (Antisense)	158
OsACs5	ACGGTTGACTCAGAAGGCTG (Sense)	TGGCTGGCGTGAATGTAGTT (Antisense)	85
EIN2	CAGTGACAACCAACTGCTGC (Sense)	GCTTGTATCGCTTCAGCACG (Antisense)	518
OsFbox072/EIN3	ACTGTCTGGTTGCTCGAAGG (Sense)	CTCCAAAGACTGCCCCATGT (Antisense)	70
OsCOI2	TCTCAAAATCGGAGAGATTTACAAG (Sense)	CGAAAGCAAAGCTCTCTCGC (Antisense)	292
OsbZIP08/TGA2	TGCTTCATGTGGCTAGGTGG (Sense)	TCCGGGTGAGGCTTCAAAAT (Antisense)	99
OsPR1#101	GCCAAGTAGACGACCACTCAC (Sense)	GAAATGTACTGCGGCAGCGA (Antisense)	73
